# DNA Methylation Profiles of Primary Colorectal Carcinoma and Matched Liver Metastasis

**DOI:** 10.1371/journal.pone.0027889

**Published:** 2011-11-21

**Authors:** Kazuo Konishi, Yoshiyuki Watanabe, Lanlan Shen, Yi Guo, Ryan J. Castoro, Kimie Kondo, Woonbok Chung, Saira Ahmed, Jaroslav Jelinek, Yanis A. Boumber, Marcos R. Estecio, Shinji Maegawa, Yutaka Kondo, Fumio Itoh, Michio Imawari, Stanley R. Hamilton, Jean-Pierre J. Issa

**Affiliations:** 1 Department of Leukemia, Division of Cancer Medicine, The University of Texas M. D. Anderson Cancer Center, Houston, Texas, United States of America; 2 Department of Pathology, Division of Pathology and Laboratory Medicine, The University of Texas M. D. Anderson Cancer Center, Houston, Texas, United States of America; 3 Division of Gastroenterology, Department of Medicine, Showa University School of Medicine, Tokyo, Japan; 4 Division of Gastroenterology and Hepatology, Department of Internal Medicine, St. Marianna University School of Medicine, Kawasaki, Japan; 5 Division of Molecular Oncology, Aichi Cancer Center, Nagoya, Japan; University of Hong Kong, Hong Kong

## Abstract

**Background:**

The contribution of DNA methylation to the metastatic process in colorectal cancers (CRCs) is unclear.

**Methods:**

We evaluated the methylation status of 13 genes (MINT1, MINT2, MINT31, MLH1, p16, p14, TIMP3, CDH1, CDH13, THBS1, MGMT, HPP1 and ERα) by bisulfite-pyrosequencing in 79 CRCs comprising 36 CRCs without liver metastasis and 43 CRCs with liver metastasis, including 16 paired primary CRCs and liver metastasis. We also performed methylated CpG island amplification microarrays (MCAM) in three paired primary and metastatic cancers.

**Results:**

Methylation of p14, TIMP3 and HPP1 in primary CRCs progressively decreased from absence to presence of liver metastasis (13.1% vs. 4.3%; 14.8% vs. 3.7%; 43.9% vs. 35.8%, respectively) (*P*<.05). When paired primary and metastatic tumors were compared, only MGMT methylation was significantly higher in metastatic cancers (27.4% vs. 13.4%, *P* = .013), and this difference was due to an increase in methylation density rather than frequency in the majority of cases. MCAM showed an average 7.4% increase in DNA methylated genes in the metastatic samples. The numbers of differentially hypermethylated genes in the liver metastases increased with increasing time between resection of the primary and resection of the liver metastasis. Bisulfite-pyrosequencing validation in 12 paired samples showed that most of these increases were not conserved, and could be explained by differences in methylation density rather than frequency.

**Conclusions:**

Most DNA methylation differences between primary CRCs and matched liver metastasis are due to random variation and an increase in DNA methylation density rather than de-novo inactivation and silencing. Thus, DNA methylation changes occur for the most part before progression to liver metastasis.

## Introduction

Colorectal cancers (CRCs) are the second leading cause of death from cancer and the third most commonly diagnosed cancers in the United States [1]. About 5% of the US population will develop CRCs within their lifetime [2]. CRCs are frequently curable by surgical resection when diagnosed at an early stage, while it is difficult to cure when patients are first seen at an advanced stage. Patients with metastatic CRCs have poor outcome with shortened survival.

Most CRCs develop in a multistep manner through the adenoma-carcinoma sequence over many years to decades [3]. The process often begins with inactivation of the APC/β-catenin signaling pathway. Accumulation of specific genetic and epigenetic events results in disease progression along three distinct clinico-pathologic pathways involving DNA methylation, microsatellite instability, and epigenetic-genetic interactions affecting mutations of KRAS or BRAF oncogenes and the p53 tumor suppressor genes [4], [5].

The molecular mechanisms responsible for progression to CRC metastasis are largely unknown. An early model postulated that metastasis results from rare molecular events that provide the ability to invade, disseminate and survive at distant sites [6] as a result of clonal selection. This model predicts that some genetic or epigenetic changes will uniquely characterize metastatic lesions as compared with their primary. Recently, gene expression studies suggested an alternative model in which the ability to metastasize is an early event that can already be distinguished even in primary tumors [7]. Altered expression of multiple genes and micro RNAs have been implicated in this process, but the molecular mechanisms underlying these alterations are unknown. Recent reports have also shown that DNA methylation has prognostic implications in CRCs [8], [9], [10], [11], [12], [13]. Patients with CRCs that are microsatellite stable and have CpG islands methylator phenotype (CIMP) tend to have a worse prognosis when compared with other molecular subtypes of CRCs. Here, we test the hypothesis that aberrant DNA methylation contributes to the metastatic process in CRCs.

## Materials and Methods

### Tissue specimens and cell lines

We examined 79 sporadic CRCs comprising 36 CRCs without liver metastasis (stage I–III) and 43 CRCs with liver metastasis (stage IV/liver recurrence). A metachronous liver metastasis was defined as a liver metastasis resected at least 12 months after resection of their primary CRCs, otherwise we considered a synchronous metastasis. Among the 43 patients, 16 had both primary CRC and matched liver metastasis available for evaluation. All tissue specimens were obtained from patients who had undergone surgery or endoscopic biopsy at the M.D. Anderson Cancer Center (n = 64) or at the Showa University Hospital (n = 15). We excluded patients who had syndromic familial predisposition (familial adenomatous polyposis or hereditary nonpolyposis colorectal cancer syndrome). Written informed consent was obtained from all study patients. Tissue collection and analyses were approved by the Institutional Review Board of the University of Texas M.D. Anderson Cancer Center and the Showa University School of Medicine.

### Tissue samples and DNA preparation

We used 95 frozen samples (79 primary and 16 liver metastatic tumors) from 79 patients with CRC. Frozen tissue samples were harvested postoperatively or endoscopically and stored at −80°C. Hematoxylin and eosin (H&E) stained slides from frozen tissue blocks were reviewed by senior pathologists to evaluate the distribution of tumor cells. Representative tumor samples contained a minimum of 80% tumor cells. When colonic biopsy specimens were obtained from patients, we used chromoendoscopy with pit pattern classification to accurately distinguish between neoplastic and non-neoplastic area in the lesion [14]. DNA was extracted from the tissue samples using standard proteinase K-phenol-chloroform methods.

A total of nine colon cancer cell lines (SW48, RKO, SW480, HCT116, LoVo, Caco2, DLD1, and SW620) were obtained from the American Type Culture Collection (ATCC, Manassas, VA). All cells were cultured in recommended medium with 10% fetal bovine serum in a humidified atmosphere containing 5% CO_2_ at 37°C. Genomic DNA was extracted from these cell lines and tissue samples using a standard phenol-chloroform method.

### Bisulfite-pyrosequencing for DNA methylation analysis

Bisulfite treatment was performed as previously described [15]. Two or 3 µl of bisulfite treated DNA were used as template for bisulfite polymerase chain reaction (PCR). We used a quantitative pyrosequencing method for all DNA methylation analysis as described previously [16], [17]. Pyrosequencing measures the methylation status of several CpG sites in a given promoter. We averaged the methylation percentage of all CpG measured, because different CpG sites show highly concordant methylation.

We evaluated the methylation status of 13 genes (MINT1, MINT2, MINT31, MLH1, p16, p14 [18], TIMP3 [19], CDH1 [20], CDH13 [20], [21], THBS1 [22], MGMT [23], HPP1 and ERα [24]), which have been reported to be altered in primary or metastatic CRCs [25]. All assays were designed to study regions within 200 base pairs upstream or downstream of transcriptional start sites. As mentioned below, eight genes were selected for validation analysis of microarray results in 12 primary tumors and matched liver metastases. Primer sequences and PCR conditions for bisulfite pyrosequencing are summarized in the Table S1.

### Methylation, mutation and definition of CIMP

For most analyses, we treated DNA methylation as a continuous variable in this study. To define CIMP, however, we converted the continuous values to categorical variables (positive/negative) defined by a methylation density greater than 15%. CIMP was defined using six genes (MINT1, MINT2, MINT31, p16, p14 and MLH1) as described previously [17]. A tumor was considered CIMP-positive if two or more of the CIMP markers demonstrated methylation. All others were defined as CIMP-negative. All mutational analysis (activating mutations in codon 12 of KRAS, BRAF codon 600 and p53 exon 2 to exon 11) were previously reported for this set of samples [4], [26], [27].

### Methylated CpG island amplification microarray (MCAM)

Methylated CpG island amplification (MCA) was performed for three primary CRCs and their paired liver metastatic samples randomly selected from the 16 paired primaries and liver metastases. One was stage IV and two had liver recurrence. A detailed protocol for MCA was described previously [28]. Microarray protocols, including labeling, hybridization and post-hybridization washing procedures were as recommended by the manufacturer and are available at http://www.agilent.com (Figure S1). Amplicons from the liver metastases were labeled with the Cy5 dye and cohybridized against amplicons from their paired primary cancers labeled with the Cy3 dye on 4×44 K promoter microarrays purchased from Agilent Technologies (Agilent, Santa Clara, CA) as described previously [29]. After hybridization preparation for array slides, arrays were scanned on an Agilent scanner and analyzed using Agilent Feature Extraction software at the M. D. Anderson Microarray Core Facility.

### Data analysis and statistics

Pyrosequencing provides a methylation level (%), which was analyzed as a continuous variable for comparison of each gene with clinicopathologic variables, and we computed mean, ranges, and 95% confidence interval (95% CI). Z-score analysis was used to normalize the methylation data of multiple genes and allow the derivation of a mean methylation score. The Z-score of methylation for each gene was calculated using the following formula: (methylation density of each sample – mean value of methylation density)/SD of methylation density. When analyzing multiple genes, we used the average of the Z-score for each gene. Differences in promoter methylation between two groups and associations between methylation and clinicopathologic characteristics were analyzed by the Mann-Whitney U test. The incidence of CIMP or gene mutation and patient characteristics were compared between tumor groups using the χ2 test or Fisher's exact test when testing small numbers of samples. All tests were two sided, and *P*<.05 was considered statistically significant.

Lowess normalization and data analysis of microarray data were performed as described previously [29]. We defined hypermethylation as normalized log_2_ ratio >1.0 (equivalent to ∼2.0-fold liver metastasis/primary tumor signal intensity) based on previous validation experiments.

## Results

### DNA methylation and mutation status in primary CRCs with or without liver metastasis

**Figure 1:**
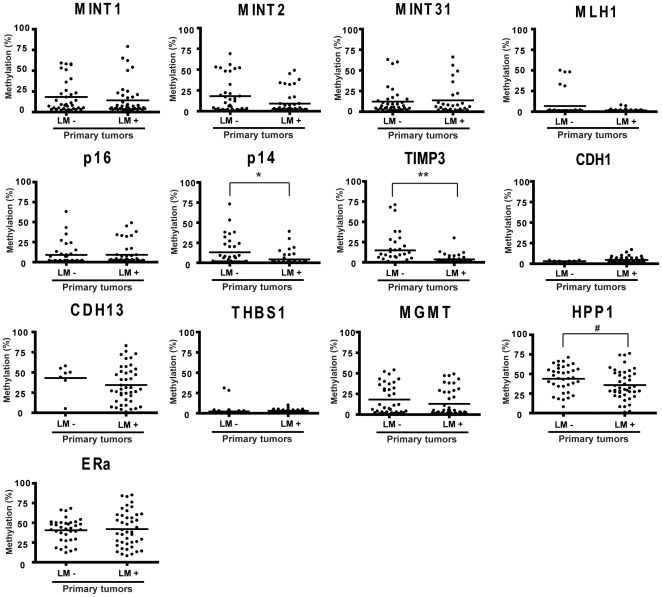
DNA methylation status of thirteen cancer-specific or age-related genes/CpG islands in primary CRCs without and with liver metastasis. Each dot represents the methylation level of an individual sample. Horizontal lines represent mean methylation levels for each group. *, *P* = .0005; **, *P* = .0113; #, *P* = .0452. LM-, primary CRCs without liver metastasis; LM+, primary CRCs with liver metastasis. CRCs, colorectal cancers.

Table 1 shows the clinicopathologic characteristics of the 79 studied CRC patients with and without liver metastasis. Of 43 CRCs with liver metastasis, 36 had synchronous liver metastasis, whereas 7 were metachronous. The 7 liver metastasis specimens were obtained 12 to 46 months after resection of primary. There were no significant differences in gender, age and tumor location between CRC patients with and without liver metastasis. Figure 1 shows the methylation status of the 13 genes investigated by bisulfite/pyrosequencing in relation to stage of primary CRC and to liver metastasis. Methylation of p14, TIMP3 and HPP1 in primary tumors progressively decreased from absence to presence of liver metastasis (13.1% [95% CI, 19.1% to 7.1%] vs. 4.3% [95% CI, 6.8% to 1.7%]; *P*<.001; 14.8% [95% CI, 21.3% to 8.2%] vs. 3.7% [95% CI, 5.3% to 2.1%]; *P* = .011; 43.9% [95% CI, 49.4% to 38.3] vs. 35.8% [95% CI, 41.6% to 30.0%]; *P* = .045, respectively). The other genes showed no significant differences by liver metastasis.

**Table 1:** Clinicopathological characteristics of patients with primary colorectal carcinomas.

		Liver metastasis	
		absence	presence
		(N = 36)	(N = 43)
Gender	Male	23	24
	Female	12	19
	Missing	1	0
Mean age	(yrs)	66.3	62.2
	(range)	(40–81)	(35–82)
Location	Proximal	15	14
	Distal	15	29
	Missing	6	0
Stage*	1	4	0
(TNM)	2	22	3
	3	8	8
	4	0	32
	Missing**	2	0
Liver metastasis	synchronous	NA	36
	metachronous		7
Non-liver	lymph node	8	30
metastasis	lung		6
	ovary	NA	2
	peritoneum		23
	brain		1

*, Stage represents initial stage when primary tumors were surgically resected. Eleven cases (three stage 2 and eight stage 3 CRCs) showed liver metastases after surgery for primary tumors.

**, Two cases were known as colorectal cancers without distant metastasis. NA, not applicable.

We next classified tumors as CIMP-positive or CIMP-negative based on methylation of 2 or more CIMP-related genes (MINT1, 2, 31, p16, p14 and MLH1) and we observed no significant difference in the frequency of CIMP between primary CRCs without and with liver metastasis (15/36, 42% vs. 13/43, 30%). When we used Z-score analysis to normalize the data of CIMP-related genes, there was no significant difference in the average of Z-scores for CIMP-related genes between CRCs without and with liver metastasis (1.5 [95%CI, 2.5 to −0.7] and 1.5 [95%CI, 2.2 to 0.7], *P* = .545). We also found no significant differences in the frequency of BRAF and KRAS mutations between primary CRCs without and with liver metastasis (3/36, 8% vs. 2/43, 5% for BRAF mutations; 19/36, 53%; 21/43, 49% for KRAS mutations). BRAF and KRAS mutations were mutually exclusive.

We also evaluated DNA methylation and mutation status of primary CRCs with synchronous and metachronous liver metastasis. Only MINT1 methylation was significantly higher in primary tumors with synchronous than those with metachronous liver metastasis (15.8% [95% CI, 22.6% to 8.9%] vs. 4.3% [95% CI, 12.9% to −0.9%]; *P* = .0121). However, we observed no significant differences in DNA methylation or mutation of the other genes between primary CRCs with synchronous and those with metachronous liver metastasis.

### DNA methylation and mutation status in matched 16 paired primary CRCs and liver metastases

**Figure 2:**
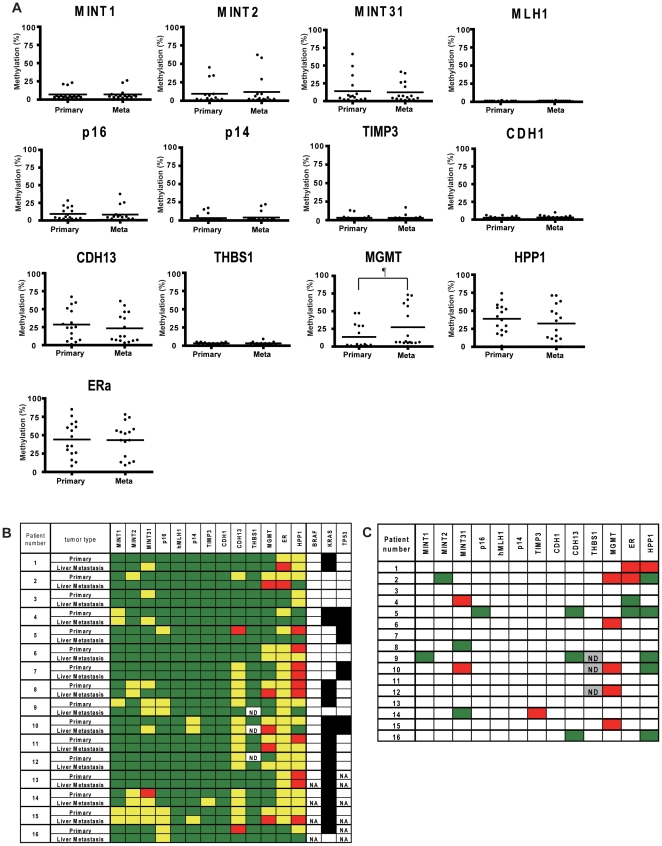
A) DNA methylation status of thirteen cancer-specific or age-related genes/CpG islands in 16 primary CRCs and matched liver metastasis. Each dot represents the methylation level of an individual sample. Horizontal lines represent mean methylation levels for each group. ¶, *P* = .013. Primary, primary CRCs; Mets, liver metastasis. B) DNA methylation and mutation status in 16 primary CRCs and paired liver metastases. Each column represents a separate gene locus indicated on top. Each row represents a primary or metastatic tumor. Average methylation density of less than 15% are shown in green, 15 to 50% in yellow and over 50% in red. Black square, presence of mutation; white square, absence of mutation; ND, not detected; NA, not applicable. C) Differences in methylation between primary CRCs and matched liver metastases. Red boxes denote an increase in methylation levels at metastatic tumors of more than two times higher when compared with primary tumors and the methylation level of at least one of the tumors is greater than 15%; green boxes show decrease of methylation levels at metastatic tumors of more than two times lower than primary tumors and the methylation level of at least one of the tumors is greater than 15%. White boxes are all others.

We measured DNA methylation for 13 genes in 16 paired primary and liver metastasis specimens which resulted in 205 measurement pairs (Data for THBS1 methylation in one primary and two metastatic tumors was not available). The data are shown in Figure 2. When analyzed as a continuous variable (Fig. 2A), only MGMT methylation was significantly higher in liver metastases than their matched primary CRCs (27.4% [95% CI, 42.6% to 12.2%] vs. 13.4% [95% CI, 22.8% to 4.2%], *P* = .013). Fig. 2B shows the data with methylation analyzed as a categorical variable. Concordant and discordant methylation between primary and metastatic tumors were respectively observed in 47 (23%) and 22 (11%) of 205 measurement pairs using a 15% cut-off value for methylation densities (Fig. 2B). A total of 183 (89%) measurement pairs showed concordant methylation status (methylation or lack of methylation). Discordant mutation was found in 2/11 (18%) tumor pairs with KRAS mutation and these two cases showed KRAS mutation in the primary tumors only (Fig. 2B). However, no discordant mutation status of p53 was observed.

We next analyzed in detail the changes in methylation levels between primary and metastatic tumors (Fig. 2C). When tumor pairs had a greater than two-fold difference in methylation level and the methylation level of at least one of the tumors was greater than 15%, we considered this a meaningful difference in methylation. Increased and decreased methylation in liver metastases were found in 11 of 205 (5%) and 14 of 205 (7%) measurement pairs, respectively. The only gene that had consistent differences was MGMT, which had increased methylation in liver metastases in 5/16 cases. However, 4 of these 5 cases showed methylation in the primary tumor as well, with an increase in methylation density in the paired liver metastasis. Of the 5 cases, 3 had synchronous liver metastases and 2 were metachronous.

### Genome-wide DNA methylation analysis in primary CRCs and matched liver metastasis

**Figure 3:**
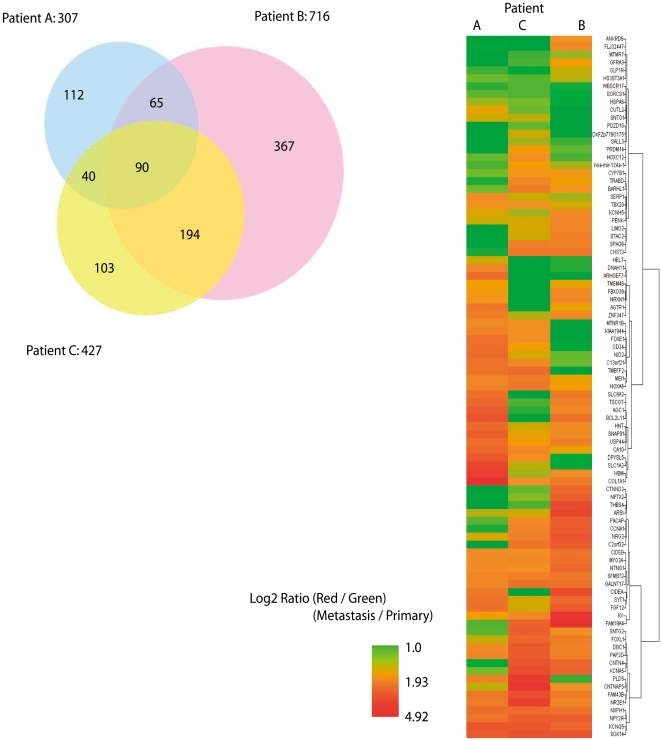
Microarray analysis of hypermethylated genes in liver metastatic cancers. A) The Venn diagram shows the overlap and differences in methylated genes of liver metastasis in three patients. A total number of 6528 genes were analyzed by 18340 microarray probes recognizing promoter CpG islands. B) Dendrogram and heat map overview of unsupervised hierarchical cluster analysis of DNA methylation in liver metastatic cancers of three patients. Each cell represents DNA methylation status of a gene in an individual sample. Red and green in cells reflect high and low methylation level, respectively, as shown in the scale bar (log_2_-transformed scale) below the matrix.

We used MCAM in three paired primary tumors and liver metastasis (Table 2). This microarray determines the methylation status of 6528 genes, of which 5940 (91%) have CpG islands within 1 kb from the transcription start sites. Figure S1 shows a representative example demonstrating gains of methylation in the metastasis sample in one case. Overall, MCAM analysis showed that 307 (4.7%), 716 (10.8%), and 427 (6.5%) genes were differentially hypermethylated in each liver metastasis sample, with 90 (1.3%) genes being commonly differentially hypermethylated in each liver metastasis (Fig. 3). Of the three tumor pairs, one was synchronous and two were metachronous metastatic tumors. Interestingly, the numbers of differentially hypermethylated genes in the liver metastatic tumors increased with increasing time between resection of the primary and resection of the liver metastasis (Table 2). These differences were statistically significant (*P*<0.0001) (Table 2).

**Table 2:** Patient's characteristics analyzed by MCAM.

Patient	Gender	Age	Tumor	Size	Histology	Liver	Duration*	Genes methylated at
		(yrs)	location	(mm)		metastasis	(months)	liver metastasis
A	F	65	Proximal	40	Mod	synchronous	3	307 (4.7%)**
B	M	73	Proximal	51	Mod-Muc	metachronous	46	716 (10.9%)**
C	M	60	Distal	23	Mod	metachronous	12	427 (6.5%)**

* , Duration between surgical resection for primary cancer and surgical resection for liver metastasis.

** , A vs. B; B vs. C; A vs. C;

p<.0001. Mod, moderately differentiated adenocarcinoma; Muc, mucinous carcinoma; NA, not applicable.

**Figure 4:**
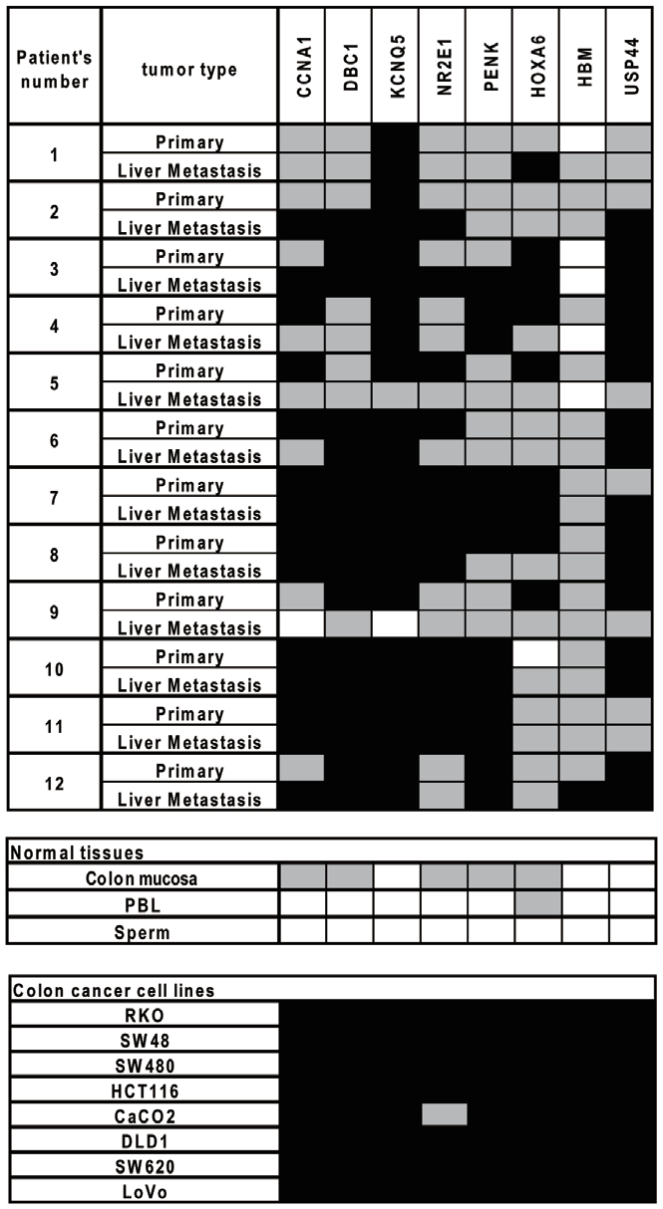
DNA methylation analysis for eight genes identified by MCAM in 12 paired primary CRCs and liver metastasis. Each column represents a separate gene locus indicated on top. Each row represents a primary or metastatic tumor, normal tissue type or colon cancer cell. Average methylation densities of less than 15% are shown in white, 15 to 50% in gray and over 50% in black. PBL, peripheral blood.

To validate the results and determine whether these changes were a result of selection or random drift with time, we selected eight hypermethylated genes that had an average log_2_ ratio value >1.9 in all 3 tumors and analyzed them by bisulfite-pyrosequencing in 12 paired primary and liver metastases of CRCs. As shown in Figure 4, all 8 genes were very commonly methylated in colon cancer cell lines and in primary tumors. In the three pairs analyzed by MCAM, 12/24 measurements showed increased methylation by pyrosequencing by our strict criteria described above, and most of the other 12 measurements also showed increased methylation (albeit to lower levels), thus validating the MCAM results. However, when studying all cases, we observed no significant differences in the methylation level of the eight genes between primary and liver metastatic cancers (Z-score: 0.116 [95% CI, 0.536 to −0.304 and −0.116 [95% CI, 0.362 to −0.595], respectively; *P* = .583) (Fig. 4). Also, these differences were not associated with time interval from resection of the primary tumor to resection of the liver metastasis. Overall, of 96 measurements, 4 measurements (4%) showed an increase, 16 measurements (17%) showed a decrease, and 76 measurements (79%) showed no change. Thus, most of the methylation differences between primary CRCs and matched liver metastasis reflect random variation rather than selection for particular genes in the metastasis process.

## Discussion

Promoter DNA methylation and associated silencing is a frequent and early event in colorectal carcinogenesis [30]. Some of the genes affected, such as MLH1, p16 and p14, clearly contribute physiologically to the neoplastic phenotype [31], [32], [33]. The occurrence of liver metastasis leads to a poor clinical outcome in CRCs, and here we sought to determine the possible involvement of DNA methylation in the process. Generally, we found that methylation does not increase with increasing stage, confirming that it is an early event. Importantly, we did find substantial drift in methylation patterns in liver metastases compared to primary tumors, but the patterns at loci examined appeared more consistent with random flux rather than selection for specific genes.

When we looked at the differences in methylation between primary tumors with and without liver metastases, methylation levels of p14, TIMP3 and HPP1 progressively decreased from early-stage to late-stage disease. We have previously found that methylation of p14 and TIMP3 is the markers for predicting CIMP1 [4]. Thus, this consistent decrease of methylation in CRCs with liver metastasis likely represents the generally good prognosis of CIMP1 cancers which rarely progress to advanced disease [11]. Depletion of TIMPs has been reported to abrogate normal apoptotic programs, enhance primary tumor growth and angiogenesis, invasiveness, and metastasis and possibly contribute to all stages of malignant progression including metastasis [34]. Our data are not consistent with a major role for TIMP3 in CRC metastasis. It is possible that other members of the TIMP family such as TIMP1 and TIMP2 might be more important for the liver metastatic process in CRCs [35].

Overall, we quantitatively compared the methylation status of 21 genes (13 candidates and 8 from the microarrays) between paired primary and liver metastasis lesions. Of these, only MGMT methylation was consistently higher in the liver metastases than primary tumors. Of 16 pairs studied, five (31%) showed significantly higher MGMT methylation at the metastatic site. Of these five tumor pairs, four pairs demonstrated MGMT methylation at both sites (primary and liver metastatic tumors) with an increase in methylation density. Increased density of methylation could be explained by multiple different factors – increased proportion of methylated cells, switch from monoallelic to biallelic methylation or even differences in the degree of normal cell contamination of the tumor samples. Our data do not allow us to distinguish these possibilities and a larger series with more detailed analysis is needed to confirm our results and address the issue.

MGMT protein stoichiometrically repairs O^6^-alkylG-DNA adducts [36]. Inactivation of MGMT by promoter-methylation can lead to G to A transition mutations in several genes, including KRAS [37]. Thus, MGMT methylation could be associated with the metastatic process by increasing the rate of mutations. However, this has not yet been convincingly demonstrated in CRCs. Park et al. have reported that MGMT methylation in patients with gastric carcinoma is significantly associated with lymph-node metastasis, tumor stage and disease free survival [38]. However, another study showed significant association between MGMT methylation and improved overall survival in diffuse large B-cell lymphoma [39]. Thus, the relationship between MGMT methylation and metastasis or tumor prognosis might be tissue specific, or possibly coincidental.

Our genome-wide analysis of hypermethylated genes at the liver metastatic tumor revealed that 7.4% (range, 4.7% to 10.9%) of the genes showed hypermethylation in the metastatic tumors and 1.3% was commonly hypermethylated among three patients. These numbers are quite large at face value, but when we validated the data by bisulfite-pyrosequencing, a change in methylation density was the explanation in most cases. One additional clue to explain this finding came from an analysis of resection time differences between the primary and metastatic lesions. Thus, the percentage of hypermethylated genes at liver metastasis was significantly higher in metachronous metastasis than in synchronous metastasis. In one patient, the time between surgery for the primary tumor and the liver metastasis was 46 months and 10.9% of genes analyzed using MCAM showed differential hypermethylation at the liver metastatic tumor. MCAM data in a patient with synchronous metastasis revealed 4.7% differential hypermethylated genes. Given that population doubling (reflected by patient age) is a prime determinant of methylation in normal and neoplastic colon, [40] our data could be explained by continued accumulation of methylation at the metastatic site. Overall, looking at methylation frequency, we find few differences between primary tumors and liver metastases, suggesting that aberrant DNA methylation is a very early event and that tumor cells acquire methylation changes before progression to liver metastasis. We cannot exclude the possibility that a few rare genes are highly selected for during the process of metastasis, but discovering these will require whole-genome methylation analysis technology that is more quantitative than what is currently available.

In summary, our results indicate that methylation frequency between primary tumors and matched liver metastasis is similar, suggesting that tumor cells acquire methylation changes before progression to liver metastasis. While we cannot rule out rare consistent changes, it appears that DNA methylation frequency is very stable over time in CRC.

## Supporting Information

Figure S1 **Scatter plot analysis of signal intensity (log scale) between DNA samples of liver metastasis (y-axis) and primary tumors (x-axis) from MCAM.**
(TIF)

Table S1 **Summary of the PCR and sequencing primers.** Primer sequences and PCR condition for MINT31, p16 and p14 were previously described.^17^
(RTF)
