# Supplementary figures and images for: DNA Methylation Profiles of Primary Colorectal Carcinoma and Matched Liver Metastasis
Source: PLoS One. 2011 Nov 21;6(11):e27889. doi: 10.1371/journal.pone.0027889 (PMC3221680; doi:10.1371/journal.pone.0027889)

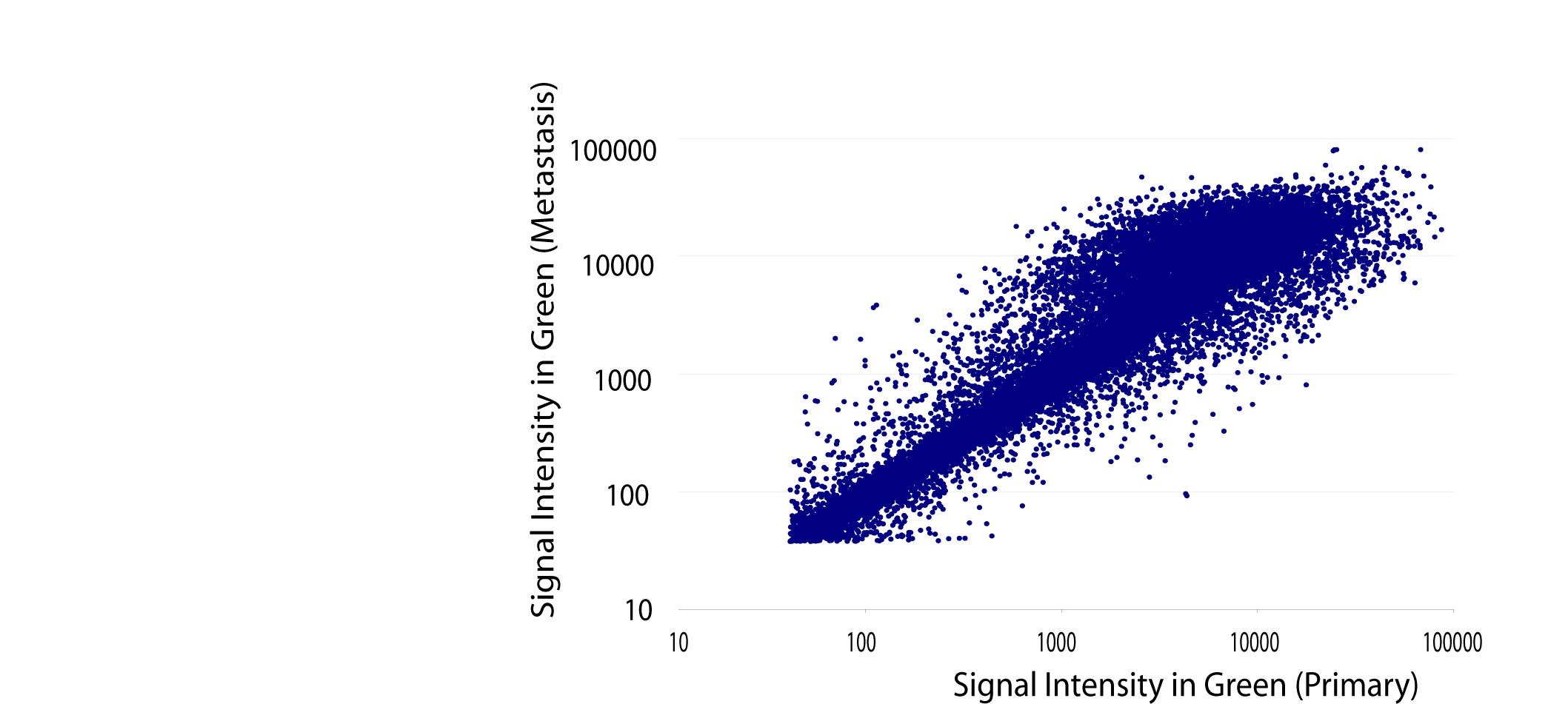

Supplement: Figure S1 — Scatter plot analysis of signal intensity (log scale) between DNA samples of liver metastasis (y-axis) and primary tumors (x-axis) from MCAM. (TIF) [file pone.0027889.s001.tif]
