# Supplementary material for: DNA Methylation Profiles of Primary Colorectal Carcinoma and Matched Liver Metastasis
Source: PLoS One. 2011 Nov 21;6(11):e27889. doi: 10.1371/journal.pone.0027889 (PMC3221680; doi:10.1371/journal.pone.0027889)
Supplement: Table S1 — Summary of the PCR and sequencing primers. Primer sequences and PCR condition for MINT31, p16 and p14 were previously described.17 (RTF) [file pone.0027889.s002.rtf]

Table S1.
Region	Primer sequences and annealing temperature	Product length (bp)	Sequencing primers	Number of CpGs	
MINT1	Forward
GGTTTTTTGTTAGYGTTTGTATTT
Reverse-Universal
GGGACACCGCTGATCGTTTAATTAATCCCTCTCCCCTCTAAACTT
50/48/46/44C	132	TTTAGTAAAAATTTTTTGGG	2
	
MINT2	Forward
AGTGTTAGAAAAATGTGTTG
Reverse-Universal
GGGACACCGCTGATCGTTTACTACAATTAAACATCAATTATATTAAACTT
50/48/46/44C	163	GAATTTTAGTATTTAAGTT	2
	
MLH1	Forward
TTGGTATTTAAGTTGTTTAATTAATAGTTG
Reverse-Universal
GGGACACCGCTGATCGTTTAAAAATACCTTCAACCAATCACCTC
50/48/46/44C	119	AGTTATAGTTGAAGGAAGAA	2
	
TIMP3	Forward
TTTTGGTTTGGGTTAGAGATAT
Reverse-Universal
GGGACACCGCTGATCGTTTACCCCCTCAAACCAATAAC
55C	276	ATTTTTTATAAGGATTTGAA	5
	
CDH1	Forward
GGAATTGTAAAGTATTTGTGAGTTT
Reverse (5'-Biotin)
TCCAAAAACCCATAACTAACC
55C	128	GGAAGTTAGTTTAGATTTTA	4
	
CDH13	Forward
TTTGGGAAGTTGGTTGGTTG
Reverse (5'-Biotin)
ACAACCCCTCTTCCCTACCT
55C	186	GGAAAATATGTTTAGTGTAG	3
	
THBS1	Forward
GGYGGAGAGAGGAGTTTAGATTG
Reverse-Universal
GGGACACCGCTGATCGTTTACCTAAACTCRCAAACCAACTC
60C	158	GGAGGAATTTTTAGGAATG	5
	
MGMT	Forward
GGTAAATTAAGGTATAGAGTTTT
Reverse (5'-Biotin)
AAACAATCTACRCATCCT
55C	156	GGAAGTTGGGAAGG	3
	
HPP1	Forward
TGTTTTTTYGTYGGGTGTTATTGTTAT
Reverse-Universal
GGGACACCGCTGATCGTTTATCCCACAACACCATAACTAATTC
50C	155	GGGATGTTTAGTAGTT	4
	
ERa	Forward
TGTGTTTTTTTTTTAGGTGG
Reverse (5'-Biotin)
AACCATCCCAAATACTTTAATA
58/56/54/52C	125	GGATACGGTTTGTATTTTG	3
	
CCNA1	Forward
GGTTGTTAGYGGTTGTTGGGAGAA
Reverse-Universal
GGGACACCGCTGATCGTTTATCTACACACRTCCCCTCTAATCC
58/56/54/52C	124	GAAATAGTTTTTTTTAAAGT	2
	
DBC1	Forward
TGGAGAATGGAGAGGGAAGTTT
Reverse-Universal
GGGACACCGCTGATCGTTTACCCCTCCCCCATTCATTTT
58C	118	TGGAGAGGGAAGTTTAAG	8
	
KCNQ5	Forward
TGGTGGAGGGGTATGGTATTATT
Reverse-Universal
GGGACACCGCTGATCGTTTACCCCCCACTCAAAAATCTCT
60C	150	GGGTTTTAAGGAGGAAGT	6
	
NR2E1	1st PCR
Forward
AGGAGTTGGGGGAAAAGTTAA
Reverse 1
AAATCCCCCAAATTCATTACC
57C
2nd PCR
Forward
AGGAGTTGGGGGAAAAGTTAA
Reverse-Universal
GGGACACCGCTGATCGTTTACCCCTCCCCCATTCATTTT
57C	145


138
	GAGATATTATAGGGGATTTAGT	3
	
PENK	1st PCR
Forward
GGAAAAGAGTAGGGTGTTTTAGGT
Reverse 1
CCCCCAAAAATACTCCTTTCT
60C
2nd PCR
Forward
GGAAAAGAGTAGGGTGTTTTAGGT
Reverse-Universal
GGGACACCGCTGATCGTTTACCCCACCCACAACTTTTAA
60C	146


112
	GAGTAGGGTGTTTTAGGTAGT	5
	
HOXA6	Forward
TAGAGTTGGATTGTTGGTAGAAATAAGG
Reverse-Universal
GGGACACCGCTGATCGTTTACCTTCTTAAACCAACTACCCCTCTA
51C	132	TAYGTTTTGTTYGGGAGATT	3
	
HBM	Forward
GGGTGTTYGGAGGTTTTATAAG
Reverse (5'-Biotin)
GGGACACCGCTGATCGTTTAGGGYGYGGTTTTTAGAGTA
58/56/54/52C	129	RTAACCCRCAATCAAATC	3
	
USP44	Forward
GGTATTTGGGGGTTTATT
Reverse (5'-Biotin)
GGGACACCGCTGATCGTTTATGAGAGAGGGAGAGTTTG
58/56/54/52C	97	CCTCRACTCATTCCCTTC	3
	
